# Jimmy Carter and Eradication of Guinea Worm Disease

**DOI:** 10.3201/eid3207.AC3207

**Published:** 2026-07

**Authors:** Shannon O’Connor, Donald Hopkins, Vitaliano A. Cama

**Affiliations:** Centers for Disease Control and Prevention, Atlanta, Georgia, USA (S. O’Connor, V.A. Cama); The Carter Center, Atlanta (D. Hopkins)

**Keywords:** Guinea worm disease, parasites, Jimmy Carter, An Everlasting Love, Sherri Richards, dracunculiasis, Dracunculus medinensis

**Figure:**
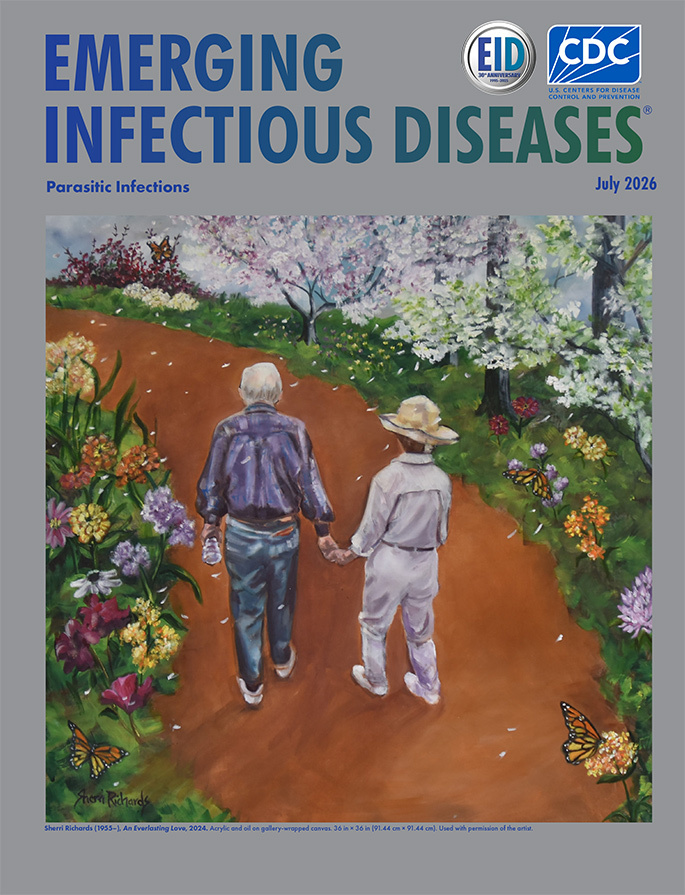
**Sherri Richards (1955–), *An Everlasting Love*, 2024.** Acrylic and oil on gallery-wrapped canvas. 36 in x 36 in (91.44 cm x 91.44 cm). Used with permission of the artist.

This month’s cover, *An Everlasting Love* by Sherri Richards, features former US President Jimmy Carter (1924–2024) and his wife, Rosalynn (1927–2023). The painting, inspired by a photograph Mrs. Richards’ husband, Dr. Frank Richards, took of the Carters on a trip to Ethiopia in 2007, depicts the Carters walking along a red dirt path that winds through a field of flowers and trees.

“The road had a reddish color, like the red clay of Georgia, and President and Mrs. Carter were walking up that road,” Mrs. Richards recalled. “[The Carters] going up the path hand-in-hand was me seeing them walking to heaven together” (S. Richards, pers. comm., interview, 2026 Apr 13).

Mrs. Richards lives in Atlanta, Georgia, USA, and holds a fine arts degree from Georgia State University. Her husband, a public health expert on the elimination of parasitic diseases, worked for The Carter Center until he retired in 2024. *An Everlasting Love* was created that same year in response to an invitation from The Carter Center to donate a painting for its annual donor retreat and auction, as she had done in previous years.

Shown from behind, in casual clothes, the Carters’ figures are instantly familiar. In the background, Mrs. Richards included some of Mrs. Carter’s favorite flowers and butterflies. *An Everlasting Love* captures the love and kindness for humanity and each other that the Carters shared, which helped lead to their founding of The Carter Center in 1982.

The Carter Center was created with a focus on human rights and peace but also established programs to eliminate or reduce certain tropical diseases because the Carters championed health as a fundamental human right. Since its founding, The Carter Center has improved the lives of millions of people affected by diseases neglected by public health systems around the world. The Carter Center’s first executive director, appointed in 1986, was Dr. William Foege (1936–2026); Carter had appointed Dr. Foege director of the then-named Center for Disease Control (CDC) in 1977, during his presidency.

In the history of public health, only 2 diseases have been globally eradicated. First was smallpox in 1980, during Dr. Foege’s tenure at CDC and in which he had a critical role; that accomplishment is heralded as one of mankind’s greatest achievements. The second was rinderpest, a devastating veterinary disease with high mortality rates in cattle and wildlife, declared eradicated in 2011. Eradication of smallpox and rinderpest permanently relieved the world of 2 deadly infectious threats. Now, thanks to the work of President Carter and The Carter Center, another centuries-old illness might soon be added to that short list: dracunculiasis, also known as Guinea worm disease.

Guinea worm disease, a tropical disease of poverty, is caused by the nematode *Dracunculus medinensis.* Humans contract infections when they drink water contaminated with copepod crustaceans infected with Guinea worm larvae. When the copepod is digested, larvae penetrate the digestive tract, migrate into the body, and develop into adults. After ≈1 year, the gravid adult female worm makes its way to the skin, where it causes a burning and painful blister. Infected people seeking relief immerse the affected limb in water (often a nearby pond), which triggers the worm to emerge and eject larvae, contaminating the water. Copepods then ingest the larvae, perpetuating the life cycle.

During worm emergence, infected people experience intense pain, ulcer formation, and allergic reactions. Other symptoms include infections of the wound, general malaise, fever, and gastrointestinal symptoms. If the worm breaks during emergence, severe allergic reactions can result. Potential long-lasting complications include chronic inflammation, pain, and, in severe cases, disability resulting from joint damage.

Dracunculiasis is an ancient disease and was first called Guinea worm disease by Europeans who saw it along the Gulf of Guinea in West Africa. The organism has been found in an Egyptian mummy and likely was referenced by the Greek writer Agatharchides (2nd Century BCE). An anonymous medieval illustration depicting Saint Roch pointing his forefinger at a worm emerging from a wound on his inner thigh probably portrays dracunculiasis (Figure).

**Figure:**
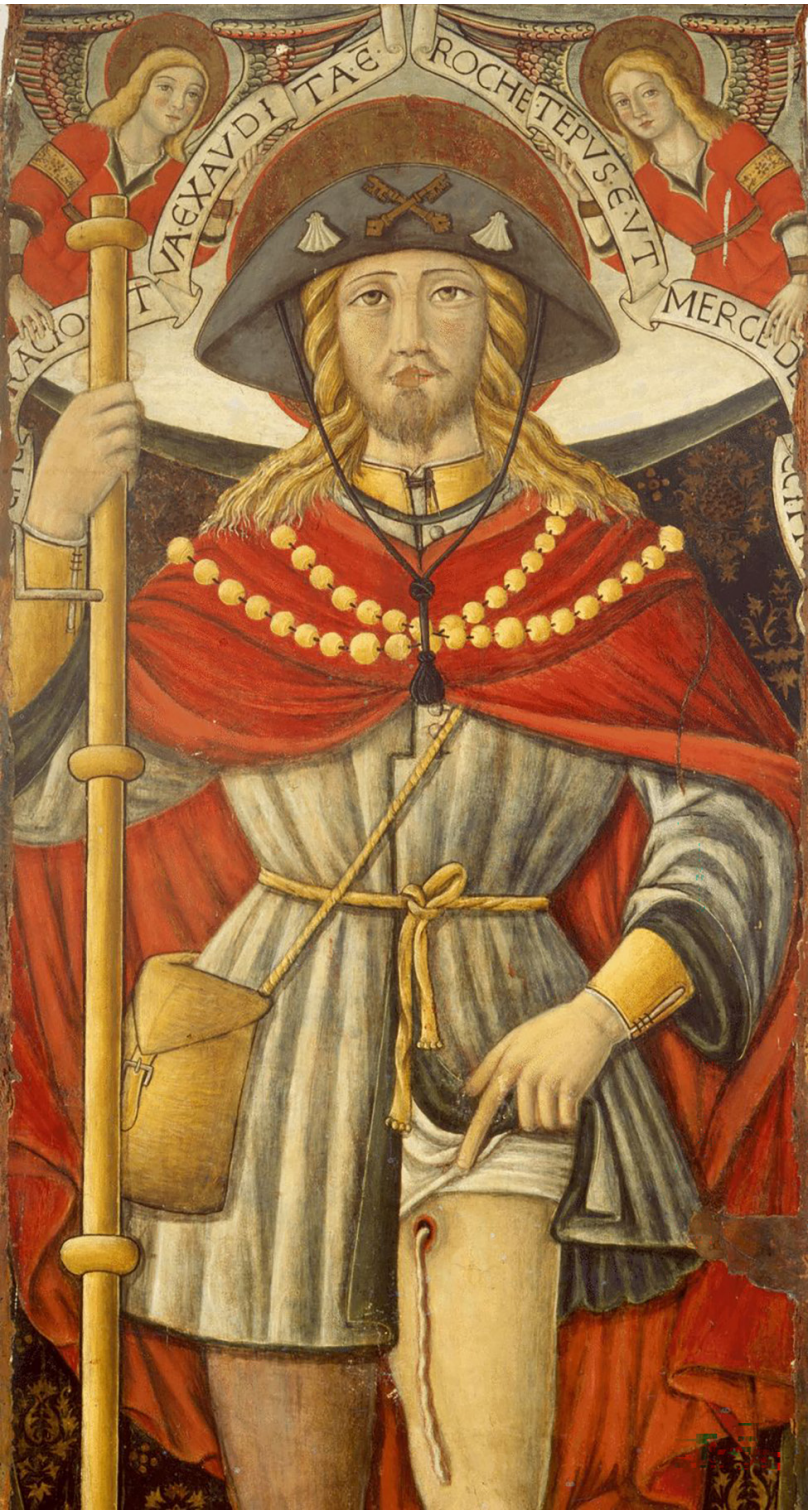
An image of Saint Roch, a French pilgrim and plague saint from the 14th Century, by an unknown painter (ca. 15th–16th Century). The painting shows what is likely a Guinea worm emerging from his leg. Source: Wikimedia Commons.

In 1980, after smallpox eradication, the Guinea Worm Eradication Program (GWEP) began at CDC. By 1982, the World Health Organization and the US Agency for International Development convened the first international meeting on Guinea worm eradication; in 1986, the World Health Assembly called for elimination of Guinea worm disease, a goal that it later upgraded to global eradication. Also in 1986, aided by Carter’s leadership and unique diplomatic skills, The Carter Center became the leading partner for the GWEP. Over the past 4 decades, CDC has provided technical support for those efforts, which have been endorsed by the World Health Organization, implemented by national programs of endemic countries, and backed by many other partners and benefactors.

The Carter Center and its partners’ ongoing work to fight Guinea worm disease has yielded astounding results. In 1986, an estimated 3.5 million human cases of Guinea worm disease occurred across 21 countries in Asia and Africa. By the end of 2025, that number had dwindled to just 10 cases in 3 countries. That dramatic decrease was accomplished without drugs or vaccines, instead focusing on clean water, behavior modification, vector control, and other nonpharmaceutical interventions. However, in 2023, parasite persistence in animals led to redefinition of worldwide eradication by the International Commission for the Certification of Dracunculiasis Eradication as “confirmed absence of the emergence of adult female worms (defined as compatible with the interruption of transmission of *D. medinensis*) in humans and animals for three consecutive years or longer at the global level.” Approximately 700 cases were detected in animals in 6 countries in 2025.

The GWEP has pioneered several interventions to support Guinea worm eradication efforts. Carter himself recruited many strategic partners that committed to support GWEP until eradication is accomplished. Those long-lasting partnerships ensured sustainability of key program interventions, such as filtering water through cloth to remove copepods, using variant straw-type filters for drinking water directly from ponds, and chemically treating water to reduce copepod infestation. In 2025, The Carter Center produced a documentary, *The President and the Dragon*, highlighting Carter’s work behind the scenes toward the goal of dracunculiasis eradication.

The Carter Center has also spearheaded peace efforts to support health initiatives. In 1995, Carter brokered a cease fire in the Sudan civil war that enabled the GWEP to work in the conflict areas, while also providing urgently needed medical support and immunizations. Those efforts became the model for other similar peace for health initiatives. On the epidemiologic side, the GWEP established a reward system to improve detection of infections and postelimination surveillance. Most of those initiatives became established thanks to direct support from Carter. The Carter Center has also established health programs for elimination of diseases including river blindness (onchocerciasis), lymphatic filariasis, malaria, schistosomiasis, and trachoma.

Late in his life, President Carter stated he hoped to outlive Guinea worm disease. He did not reach that milestone, but his long years of support for interventions and research helped bring Guinea worm eradication within reach. When he accepted the Nobel Peace Prize in 2002, Carter said, “The bond of our common humanity is stronger than the divisiveness of our fears and prejudices. God gives us the capacity for choice. We can choose to alleviate suffering.” Long after President Carter’s death, the Carters’ legacy at The Carter Center will continue to alleviate suffering and advance public health by fighting to eradicate Guinea worm disease and other neglected diseases.
